# Effects of different frozen temperatures of pork sausage batter on quality characteristics of reduced-salt sausages using pre-rigor muscle

**DOI:** 10.5713/ab.21.0421

**Published:** 2022-01-05

**Authors:** Geon Ho Kim, Koo Bok Chin

**Affiliations:** 1 Department of Animal Science, Chonnam National University, Gwangju 61186, Korea

**Keywords:** Pre-rigor Muscle, Pork Sausage, Quality Characteristics, Reduced-salt

## Abstract

**Objective:**

The objective of this study was to evaluate quality characteristics of reduced-salt pork sausage (PS) using pre-rigor muscle compared to those of regular-salt PS. In addition, effects of freezing on sausage batter with different temperatures (−30°C vs −70°C) on quality characteristics of both sausage batter and cooked sausages during frozen storage were observed.

**Methods:**

Pre-rigor and post-rigor pork hams were used to manufacture low-fat sausages. Sausages using post-rigor (Post) muscle were manufactured at a salt level of 1.5%, whereas those with pre-rigor (Pre) muscle were processed at salt level of 1.0%. After these muscles were made at two salt levels (1.5% salt, Post-rigor; 1.0% salt, Pre-rigor), Sausage batters were stored at two frozen temperatures (−30°C vs −70°C). During storage for 12 wks, they were measured for physicochemical and textural properties every 4 wks up to 12 wks.

**Results:**

pH values and temperatures of sausage batter of pre-rigor muscle were higher than those of post-rigor muscle regardless of the frozen temperature. The lightness and yellowness values of batter at the initial storage were the highest during storage. For PS, there were no differences in most parameters measured among all treatments. However, expressible moisture values (%) of Pre-30 and Pre-70 were lower than those of Post-30 (p<0.05).

**Conclusion:**

Regardless of frozen temperature during storage, quality characteristics of pre-rigor PS with salt level of 1.0% salt were similar to those of post-rigor PS with salt level of 1.5%. By using the pre-rigor muscle, salt content could be reduced by one third of the regular-salt level (1.5%) of post-rigor muscle.

## INTRODUCTION

Sodium chloride (Salt, NaCl) is a key ingredient that performs many important functions in meat products. It is an important factor that can inhibit growth of microorganisms by decreasing water activity [1]. Since salt can inhibit the growth of microorganisms and extend the shelf-life, it is considered as an important food preservative for food safety [2]. In addition, it is essential for developing flavor of meat products [2]. Protein solubility and extractability can also be increased by the addition of salt [3]. Although salt plays a variety of key roles in meat product manufacturing, when excessive amount of salt is consumed, blood volume and blood pressure may increase, leading to cardiovascular diseases or high blood pressure [4]. Despite the need to the reduction of salt, it might cause undesirable quality of meat products. Therefore, strategies for development of acceptable reduced-sodium meat products are highly needed.

Hot boning is a kind of deboning method to separate the muscle when the carcass still has a high temperature, unlike the normal deboning method. Compared to post-rigor muscle, pre-rigor one had better functionalities such as extractability of myofibirillar proteins, binding ability, and water-holding capacity (WHC), resulting in the development of reduced-sodium meat products by improving the undesirable functionalities of those due to the reduction of salt. Puolanne and Terrell [5] reported that emulsified sausage using pre-rigor muscle maintained physicochemical and sensory properties even if the addition level of salt is reduced. In addition, Claus and Sørheim [6] reported that pH values of beef patty manufactured with pre-rigor muscle had better protein solubility, cohesiveness, and chewiness with a lower cooking loss than those of post-rigor one due to higher pH of raw meat.

The use of pre-rigor muscle for the manufacture of sausage might be compensated for defects of low or reduced-sodium sausages, even when the sausage is manufactured with frozen-stored sausage batter. Since the correlation between high pH and functionalities are highly associated with processing aptitude of pre-rigor muscle, the application of frozen storage of sausage batter could be applicable. Thus, physicochemical properties of sausage batter as affected by different frozen temperatures and time should be determined to confirm the potential use of frozen storage. Thus, the objective of this study was to evaluate quality characteristics of pork sausages (PS) manufactured with pre-rigor muscle for the development of reduced-salt sausages. In addition, whether frozen storage at two different temperatures (−30°C vs −70°C) of reduced-salt PS using pre-rigor muscle could maintain quality characteristics during 12 wks (4 wks interval) of frozen storage was also determined.

## MATERIALS AND METHODS

### Experimental design

Table 1 shows ingredients added and formulation of manufactured PSs for this study. Two rigor states (pre-rigor vs post-rigor) of raw meat and different additional levels of salt (1.0% for pre-rigor muscle and 1.5% for post-rigor one) of sausage batter were used for the quality of PS. Sausages using post-rigor muscle were prepared by adding 1.5% salt as a reference, whereas others were manufactured using pre-rigor one with 1.0% salt as a treatment. Prepared sausage batters, which were stored frozen at two different frozen temperatures (−30°C vs −70°C), were investigated the quality characteristics of PS. Therefore, there were four treatments: Post-30 (Post-rigor, 1.5% salt, −30°C), Post-70 (Post-rigor, 1.5% salt, −70°C), Pre-30 (Pre-rigor, 1.0% salt, −30°C), and Pre-70 (Pre-rigor, 1.0% salt, −70°C).

### Preparation for sausage manufacture

Fresh pork hams of castrated three-way crossbreeding pig (Yorkshire×Landrace×Duroc, Korean, 1st grade) were purchased from a local meat market (Hyundai distribution, Gwangju, Korea) and used to manufacture PSs. Post-rigor muscle (slaughtered and stored for more than 24 hrs) and pre-rigor muscle (less than 1 h after slaughtering) were prepared for manufacturing sausages. After external fat and connective tissues were removed, they were comminuted with curing agent in a meat chopper (M-12S; Hankook Fujee Industries Co., Ltd, Hwaseong, Korea) to manufacture sausages.

Raw meat and ice water were mixed with a grinder (Crypto peerless K55; DITO SAMA, Birmingham, France) for 30 s. Curing agents including salt, sodium nitrite, sodium erythorbate, and sodium phosphate were mixed for 1 min with ground pork ham and ice water. The remained ice water was added and ground twice for 30 s each to make sausage batters which were stored either at −30°C or −70°C for 12 wks in a freezer. Frozen sausage batter were thawed at 10°C in a refrigerator one day ahead of time at the processing day. They were stuffed into polyvinylidene chloride (PVDC), cooked in water bath (WB-22; Daihan Scientific Co., Ltd., Seoul, Korea) at 75°C for 30 min to reach an internal temperature of 71°C, and then cooled down in an ice water.

### Experimental methods

#### Determination of pH and color

The pH was measured with a solid pH-meter (Model 340; Metter-Toledo, Schwarzenbach, Switzerland). Mean pH values were calculated from five times for each sample. Color measurements including lightness (CIE L*), redness (CIE a*), and yellowness (CIE b*) of the cross section of each sausage sample were measured by a CIE color meter (CR-10; Minolta Co. LTD, Tokyo, Japan). Results were expressed as the calculated average of values measured five times (CIE values of the standard plate were L* = 94.8, a* = 1.0, and b* = 0.1).

#### Protein solubility

A 30 g of sausage batter and 90 mL salt solution (3% salt, 17.8 mM STPP, 1 mM NaN_3_, pH 6.0) were homogenized for 30 s. After the mixture was stored in a refrigerator for 1 hr to extract the protein, the mixture was centrifuged at 12,000×g for 1 hr. Crude protein content (%) in the supernatant of the centrifuged sample was determined with the Kjeldahl method. Protein solubility was calculated as follows:


$$\text{Protein solubility }(\%)=\frac{(Protein\;content\;of\;supernatant\;)\times 4}{Protein\;content\;of\;sausage\;batter}\times 100$$

#### Texture profile analysis

Texture profile analysis was performed to determine the hardness (gf), springiness (mm), chewiness, and cohesiveness using an Instron Universal Machine (Model 3344; Canton, MA, USA). A load cell of 50 kg was equipped with a compression probe. A two-bite test was conducted at a cross speed of 300 mm/min. These results were expressed as the average of values measured 10 times.

#### Cooking loss

Cooking loss (CL, %) of a cooked sausage was measured based on the weight of the sausage before and after cooking using the formula shown below:


$$\text{Cooking loss }(\%)=\frac{\left(Samples\;weight\;before\;cooking-Sample\;weight\;after\;cooking\right)}{Sample\;weight\;bofore\;cooking}\times 100$$

#### Expressible moisture

Expressible moisture (EM, %) of sausage was measured with the centrifugation method. Briefly, PS samples wrapped with filter paper (Whatman #3; GE healthcare, Little Chalfont, UK) were centrifuged at 1,660×g for 15 min. The EM (%) released from each sample wrapped with the filter paper was measured and derived using the following formula:


$$\text{Expressible moisutre }(\%)=\frac{Expressible\;water\;weight\;of\;filter\;paper}{Sample\;weight}\times 100$$

#### Thiobarbituric acid reactive substances

Thiobarbituric acid reactive substances (TBARS) was measured the amount of malondialdehyde (MDA) which reacted with thiobarbituric acid (TBA) in sausage according to the method of Sinnhuber and Yu [7]. Briefly, a mixture of TBA solution (3 mL) and homogenized sample (2 g) was mixed with TBA solution (17 mL) and heated in a water bath (Whatman #3; GE healthcare, UK) at 100°C for 30 min. Then, each sample (5 mL) and chloroform (5 mL) were mixed and centrifuged at 1,660×g for 5 min. The supernatant of the centrifuged sample (3 mL) was then mixed with petroleum ether (3 mL) and centrifuged again at 1,660×g for 10 min. Absorbance of reactive substance in the lower layer was then measured with a spectrophotometer (Model UV-1601; Shimadzu, Kyoto, Japan) at wavelength of 532 nm. TBARS values were derived with the following formula.


$$\text{TBARS value }(\text{mg of MDA}/\text{kg of sample})=\frac{Absorbance\;value\times 9.48}{Sample\;weight}$$

#### Volatile basic nitrogen

Volatile basic nitrogen (VBN) values were measured using the method of Conway [8] to determine protein degradation. After a homogenized sample was mixed with 9 mL of double distilled (dd)-water using a homogenizer (T-25 basic; IKA Labortechnik, Staufen, Germany) for 1 min, the mixture was then filtered using a filter paper (Whatman #2; GE healthcare, UK). And then 1 mL of the sample was dispensed into the outer chamber of a Conway cell and 1 mL of a 0.01 N boric acid solution and 50 μL of an indicator (0.066% methylene red + 0.066% bromocresol green) were dispensed into the inner chamber. Then, 1 mL of saturated K_2_CO_3_ solution (50%) was injected into the outer chamber. Conway cell was quickly sealed, horizontally agitated, and incubated at 37°C for 120 min. After incubation, 0.01 N HCl solution was titrated to the sample. The VBN value was then determined using the following formula.


$$\text{VBN value }(\text{mg }\%)=\frac{Titrated\;amount\;of\;0.01N\;HCl\;solution\;(mL)\times 0.14\times dilution\;factor\times 100}{Sample\;weight}$$

### Statistical analysis

The whole experiments were performed in triplicate. Results were expressed as means and standard deviations. All statistical analyses were performed using SPSS software program version 25.0 (SPSS Inc., Chicago, IL, USA). Statistical processing was carried out with two-way analysis of variance (ANOVA) using treatment groups (salt level and frozen temperature combinations) and frozen storage periods of sausage batters as factors. Since results of all experimental parameters had no interactions between treatments and frozen storage periods (p>0.05), data were pooled by frozen storage periods and treatments. Duncan’s multiple range test was performed as post ANOVA at significance level of 0.05.

## RESULTS AND DISCUSSION

### pH values and temperature of raw meat

Table 2 shows pH values and temperature of raw meat state used to prepare PSs. As expected, pH values and temperatures of pre-rigor muscle were higher than those of post-rigor one (p<0.05). Since pre-rigor muscle were deboned from carcass prior to rigor mortis, they had higher pH and temperature values than post-rigor muscle. According to Kim and Chin [9], pH values and temperatures of pre-rigor pork ham muscle were 6.06 and 29.2°C, respectively, whereas those of post-rigor muscle were 5.69 and 11.0°C, respectively. According to the previous study [9], pre-rigor muscle had higher pH and temperature values than post-rigor ones, similar to results of this study. Channon et al [10] reported that pH values and temperature of pork carcasses stunned with electrical stimulation were decreased from 40 min to 24 hr post slaughter. Since pre-rigor muscle did not go through postmortem, it had higher pH and temperature than post-rigor one, and there was a metabolism causing a change in rigor state. During postmortem, pH decline is caused by glycolysis and the accumulation of lactate and hydrogen ions from the absence of the circulatory system due to the lack of oxygen [11].

### pH and color values of raw sausage batters

As shown in Table 3, pH values of sausage batters using pre-rigor muscle were higher than those of post-rigor muscle. However, there were no differences in pH values between treatments within a same rigor state (p>0.05). This seems to be partially due to differences in pH values of raw meat state since the pre-rigor had normally higher pH than the post-rigor. Raw sausage batters manufactured with pre-rigor muscle had higher pH than those manufactured with post-rigor muscle, which was similar to pH trend in raw meat. Thus, sausages manufactured with pre-rigor muscle having high pH values would have higher pH than those manufactured with post-rigor muscle having lower pH, even after curing. However, frozen storage did not affect pH values of the sausage batter, regardless of the frozen temperature (p> 0.05). Park et al [12] reported that pH values of fresh pork meat did not change during frozen storage at −10°C (0, 30, 60, 90, and 120 d), regardless of difference in retail cut (belly vs. loin) or packaging method (vacuum vs aerobic). These results indicated that frozen storage might not affect the pH vales of sausages before cooking (p>0.05).

Table 3 shows color values of raw sausage batters before cooking. There were no differences in any color parameters (CIE L*, a*, and b*) among treatments. CIE L* and b* values of sausage batters prior to freezing were higher than those of sausage batters after frozen storage for 4, 8, and 12 wks (p< 0.05). However, CIE a* values of sausage batters prior to freezing were lower than those after frozen treatments (p< 0.05). These results indicated that frozen storage might affect color values of raw sausage batters. The reason for such changes in color values of sausage batters after frozen storage might be due to depletion of cofactors. They would inhibit the change from myoglobin to metmyoglobin as oxidation progresses, resulting in reduced color stability and darkening [13].

### Protein solubility of raw sausage batter

Protein solubility results of raw sausage batters are shown in Table 3. There were no differences in protein solubility among all treatments (p>0.05). In general, it is known that meat protein with higher additional level of salt has higher protein solubility. Choi et al [14] reported that protein solubility of preblended pork for frankfurter added with the higher salt level (3.0%) was higher than that of treatment with a lower level (1.5%) of salt regardless of phosphate level. However, differences in protein solubility of sausage batter between salt levels of 1.0% and 1.5% were not observed due to different rigor state in present study, resulting in similar protein solubility. Pre-rigor meat might have higher protein solubility than post-rigor one due to higher pH and ATP level, and functional properties such as water holding and emulsifying capacities of meat products using pre-rigor meat are also better than those using post-rigor one [15]. The solubilization of protein depends on pH, ionic strength and temperature, and it is a process that includes the solvation, dissolution, wetting, and swelling of the protein molecule. It indicated that functionalities are greatly affected by protein solubility [16]. Therefore, meat protein using pre-rigor state might lead to acceptable processing quality even with a less salt level (1.0%).

### pH and color values of cooked pork sausage

Table 4 shows pH and color values of cooked PSs as affected by salt content and rigor state of raw meat during different frozen temperature. No differences in pH values of cooked PSs using pre-rigor and post rigor muscle at two different salt levels were observed between different frozen temperatures and storage period (12 wks) of raw sausage batters (p> 0.05). Although pH values of cooked PSs using pre-rigor batters stored frozen at −70°C (Pre-70) were higher than those of using post-rigor raw batter (Post-30 and Post-70), pH values of cooked sausage using pre-rigor batter stored frozen at −30°C (Pre-70) were not different from those of Post-30 and Post-70 (p>0.05). Pre-70 had a higher pH than sausage groups manufactured with post-rigor muscle due to sausage batter from pre-rigor muscle with higher pH values. Miller et al [17] reported that pH values of pork loin stored at −17.8°C for 37 wks were not changed during frozen storage, resulting in maintaining pH values of muscle during frozen storage.

Color values of cooked PSs as affected by different rigor state, salt level, and frozen temperatures are shown in Table 4. These results indicated that the combination of rigor state and salt content did not affect color values of PS (p>0.05), as supported by Villamonte et al [18] who reported that chopped pork meat added with 1.5% and 3.0% of salt had similar lightness and redness values under the same additional level of phosphate. No differences in color values of PSs among different frozen storage periods of batters were also observed (p>0.05). Van Laack et al [19] observed increases in redness values of cooked beef patties after 1 yr of storage at −27°C. However, they did not explain the relationship between color change and frozen storage, which showed a different trend from the present study. The mechanism of color changes of cooked meat products during frozen storage is not fully understood yet, although many factors such as cooking methods and amount of metmyoglobin are known to affect color values. Thus, further research will be needed to understand fully how these factors affect color values of meat products [20].

### Texture profile analysis of cooked pork sausages

As shown in Table 5, no differences in textural properties of cooked PSs among treatments were observed (p>0.05). Generally, meat products with higher additional levels of salt tend to have higher textural properties than those with lower salt contents. Thus, the reduction of salt content might negatively affect textural properties of meat products. Jiménez-Colmenero et al [21] reported that low-fat cooked pork batter added with 2.5% of salt had higher textural properties than those added with 1.5% of salt. However, the addition levels of salt did not affect textural properties in the present study (p>0.05). This might be due to differences in rigor states of raw meat to use in the manufacture of PSs, suggesting that meat products using pre-rigor muscle would show higher textural properties. Lee et al [22] reported that hardness, cohesiveness, gumminess, and chewiness of low-fat hot-boned pork gel was higher than those of chilled-boned one with the same fat content. Despite differences in addition levels of salt, the rigor state might affect textural properties, resulting in similar textural properties with a combination effect. However, no changes in textural properties of cooked sausages were observed during the frozen storage period for 12 wks in the present study (p>0.05). According to a study of Kim and Chin [9], hardness and gumminess of PS using raw meat stored under frozen were lower than those using fresh meat due to muscle denaturation during frozen. It is generally accepted that during the process of freezing and thawing, myofibrillar protein is denatured, leading to deformation of existing regular and continuous microstructure [23]. In contrast, an interesting finding in this study was that frozen storage of raw sausage batter did not affect textural properties of cooked sausage, regardless of the frozen temperature and time.

### Cooking loss and expressible moisture of cooked sausages

As shown in Table 6, there were no differences in Cooking loss (CL, %) of cooked PSs among treatments (p>0.05). This result could be explained by the fact that reduced-salt sausage products were redeemed with pre-rigor muscle in terms of WHC. Kang et al [24] reported that CLs of raw sausage batters added with 2% salt were higher than those added with 1% salt. It indicated that CLs of PSs with lower salt contents (≤1.0%) were higher than those with higher salt contents because salt increased WHC and decreased CLs in meat products. The addition of sodium chloride caused swelling of myofibrillar proteins by increase negative charges [4]. This means that the side chains of the amino acids in net charge of proteins bind water molecules by ionic strength of chloride. Despite different addition levels of salt, all treatments had similar CLs due to different rigor state, which could compensate for reduced-sodium sausage products. Hamm [25] observed that CLs of homogenize beef neck muscle with salt tended to be increased as the pH declines from 6.8 to 5.4 until rigor mortis. It indicated that pH fall could occur to decrease WHC of muscles, and as the pH of meat reaches the isoelectric point (pH 5.4), the WHC of muscle decreases due to net charge effect of protein between positive and negative charges [26]. This study indicated that pre-rigor muscle could have higher WHC based on initial high pH values of the rigor state. Although CLs of PSs with a lower salt level were increased, when pre-rigor muscle were used, then they might compensate for the water loss of muscle in low or reduced-salt sausages during cooking and storage.

Expressible moisture (EM, %) results of cooked sausages as affected by salt content, rigor state, and different frozen conditions of sausage batters are shown in Table 6. No differences in EM (%) values of PSs were observed among treatments (p>0.05). There was no difference in EM values between Pre-30 and Pre-70 (p>0.05). Post-70 with a higher salt level (1.5%) and Pre-30 and Pre-70 with a reduced salt level (1.0%) had similar EM values (p>0.05). These results indicated that PSs manufactured using pre-rigor muscle with reduced-salt level had EM similar to those of Post-70 (post-rigor) with a high salt content (1.5%). Although PSs added with a high salt content had higher WHC in a previous study [27], no differences in EM between Post-30 or Post-70 with 1.5% salt and Pre-30 with 1.0% salt were observed. Honikel and Hamm [28] reported that WHC of pre-rigor muscle was higher than those of post-rigor ones as measured by filter paper press method. There were no differences in EM of cooked sausages according to the frozen storage period of sausage batters before heating (p>0.05). These results suggested that frozen storage of sausage batter might not affect WHC of the final products, if both rigor-state and salt combination were applied properly in the manufacture of processed meats.

### Thiobarbituric acid reactive substances of cooked sausages

Table 6 shows TBARS results of cooked sausages with different rigor states of raw meat, salt content, and frozen storage temperature of raw batter. No differences in TBARS values among all treatments during storage were observed (p>0.05). This indicated that different salt contents, rigor states of raw meat, and frozen conditions of raw batter did not affect TBARS values of cooked sausages. Drerup et al [29] reported that TBARS values of pre-rigor fresh PSs were lower than those of post-rigor PSs with the same salt level during storage at 0°C. This suggested that TBARS were highly related to the pH of raw meat. There was a slow lipid oxidation in pre-rigor processing. Unlike the previous study, the use of pre-rigor muscle and its pH value did not affect TBARS of PSs during frozen storage, regardless of storage temperature.

### Volatile basic nitrogen (mg%) of cooked sausages

As shown in Table 6, no differences were observed in VBN values of PSs prepared using rigor states of raw meat added with different salt contents during frozen storage (p>0.05). In a previous study of Kim and Chin [9], VBN values of PSs prepared with pre-rigor muscle added with 0.5%, 1.0%, and 1.5% of salt levels were not different from those of sausages using post-rigor one (1.5% salt). These results suggested that the no differences in VBN values were partially due to proper combination effect of salt level (1.0% vs 1.5%) and the rigor state of raw meat (pre-rigor vs. post-rigor muscle). Kim et al [30] reported that there was no difference in VBN between fresh pork loin and frozen-and-thawed one during storage. These results indicated that freezing and thawing of raw sausage batters did not affect VBN values of final sausage products, if frozen conditions of sausage batters were controlled properly.

## CONCLUSION

Although pre-rigor PSs contained the lower salt level (1.0%), most of their quality characteristics were similar to those of post-rigor PSs with the higher salt level (1.5%). It indicated that pre-rigor pork ham would have great processing aptitude based on high pH for the manufacture of reduced-sodium meat products. In addition, different frozen storage temperatures (−30°C vs −70°C) of raw sausage batter had no effect on quality characteristics of pre-rigor PSs. Therefore, pre-rigor muscle and frozen storage of sausage batter might be useful in the improvement of quality characteristics in the processing of reduced-sodium meat products.

## Figures and Tables

**Table 1:** Formulation of pork sausages with different salt level, rigor state and frozen conditions

Ingredients (%)	Treatment			

	Post-30	Post-70	Pre-30	Pre-70
Meat	80.0	80.0	80.0	80.0
Water	18.5	18.5	18.5	18.5
Non meat ingredients	2.00	2.00	2.00	2.00
Sodium chloride	1.30	1.30	0.80	0.80
Sodium tripolyphosphate	0.40	0.40	0.40	0.40
Sodium erythorbate	0.05	0.05	0.05	0.05
Cure blend^1)^	0.25	0.25	0.25	0.25
Total	100.5	100.5	100.0	100.0

1) Cure blend consisted of 93.75% of salt and 6.25% of sodium nitrite.

**Table 2:** pH and temperature of raw meat as affected by different rigor state

Item	Post-rigor	Pre-rigor
pH	5.66±0.10^b^	6.20±0.09^a^
Temperature	17.7±5.73^b^	33.8±2.22^a^

a,b Means having the different superscripts in the same row are different (p<0.05).

**Table 3:** pH, color values, and protein solubility of raw sausage batter as affected by different salt level, rigor state and frozen conditions

Item		pH	CIE L^*^	CIE a^*^	CIE b^*^	Protein solubility
Treatment^1)^						
Salt 1.5%	Post-30	5.99±0.07^b^	56.8±0.99^a^	5.80±1.94^a^	4.89±1.02^a^	54.9±12.4^a^
	Post-70	5.95±0.05^b^	56.8±1.08^a^	5.80±1.69^a^	5.09±0.70^a^	58.8±8.75^a^
Salt 1.0%	Pre-30	6.26±0.13^a^	57.7±1.39^a^	6.50±1.83^a^	5.33±1.12^a^	50.5±7.64^a^
	Pre-70	6.28±0.15^a^	57.7±1.87^a^	6.29±1.91^a^	6.01±1.00^a^	54.0±5.63^a^
Storage time (wk)						
0		6.16±0.20^a^	58.7±0.92^a^	3.40±1.04^b^	6.99±1.73^a^	58.8±12.4^a^
4		6.15±0.18^a^	56.2±1.22^b^	6.94±1.80^a^	5.48±1.52^b^	54.2±6.26^a^
8		6.13±0.20^a^	57.0±1.13^b^	6.47±1.09^a^	5.59±1.09^b^	54.2±5.28^a^
12		6.14±0.19^a^	57.1±1.10^b^	6.59±0.90^a^	5.45±1.05^b^	50.9±4.47^a^

1) 1.5%, Post-30, pork sausage (PS) with 1.5% of salt using post-rigor muscle during batter storage at −30°C; 1.5%, Post-70, PS with 1.5% of salt using post-rigor muscle during batter storage at −70°C; 1.0%, Pre-30, PS with 1.0% of salt using pre-rigor muscle during batter storage at −30°C; 1.0%, Pre-70, PS with 1.0% of salt using pre-rigor muscle during batter storage at −70°C

a,b Means having the different superscripts in the same column (treatment or storage time) are different (p<0.05).

**Table 4:** pH and color values of cooked pork sausages as affected by different salt level, rigor state and frozen conditions

Item		pH	CIE L^*^	CIE a^*^	CIE b^*^
Treatment^1)^					
Salt 1.5%	Post-30	6.21±0.05^b^	72.7±1.24^a^	10.5±0.53^a^	4.26±0.30^a^
	Post-70	6.21±0.05^b^	73.1±1.06^a^	10.5±0.58^a^	4.16±0.26^a^
Salt 1.0%	Pre-30	6.34±0.27^ab^	72.6±0.64^a^	11.1±0.50^a^	4.99±0.64^a^
	Pre-70	6.45±0.11^a^	72.7±0.91^a^	10.9±0.44^a^	5.05±0.70^a^
Storage time (wk)					
0		6.30±0.17^a^	73.4±0.70^a^	10.6±0.68^a^	4.61±0.69^a^
4		6.34±0.14^a^	73.1±0.81^a^	10.5±0.37^a^	4.74±0.69^a^
8		6.25±0.26^a^	72.3±0.65^a^	10.8±0.43^a^	4.50±0.71^a^
12		6.32±0.11^a^	72.4±1.03^a^	11.0±0.59^a^	4.61±0.56^a^

1) 1.5%, Post-30, pork sausage (PS) with 1.5% of salt using post-rigor muscle during batter storage at −30°C; 1.5%, Post-70, PS with 1.5% of salt using post-rigor muscle during batter storage at −70°C; 1.0%, Pre-30, PS with 1.0% of salt using pre-rigor muscle during batter storage at −30°C; 1.0%, Pre-70, PS with 1.0% of salt using pre-rigor muscle during batter storage at −70°C.

a,b Means having the different superscripts in the same column (treatment or storage time) are different (p<0.05).

**Table 5:** Texture properties of cooked pork sausages as affected by different salt level, rigor state and frozen conditions

Item		Hardness	Springiness	Gumminess	Chewiness	Cohesiveness
Treatment^1)^						
Salt 1.5%	Post-30	4,418±493^a^	6.18±0.72^a^	36.6±4.75^a^	220±33.4^a^	0.01±0.00^a^
	Post-70	5,015±519^a^	5.96±0.76^a^	49.7±16.1^a^	290±79.5^a^	0.01±0.00^a^
Salt 1.0%	Pre-30	4,740±821^a^	5.40±0.49^a^	45.7±12.4^a^	244±73.1^a^	0.01±0.00^a^
	Pre-70	4,853±736^a^	5.36±0.72^a^	44.3±13.3^a^	247±77.5^a^	0.01±0.00^a^
Storage time (wk)						
0		4,825±443^a^	5.70±0.35^a^	42.9±4.99^a^	243±26.5^a^	0.01±0.00^a^
4		4,934±499^a^	5.63±0.95^a^	50.4±9.97^a^	273±65.7^a^	0.01±0.00^a^
8		4,530±858^a^	5.33±0.46^a^	38.3±8.45^a^	215±58.3^a^	0.01±0.00^a^
12		4,738±816^a^	6.23±0.84^a^	44.8±20.8^a^	269±104^a^	0.01±0.00^a^

1) 1.5%, Post-30, pork sausage (PS) with 1.5% of salt using post-rigor muscle during batter storage at −30°C; 1.5%, Post-70, PS with 1.5% of salt using post-rigor muscle during batter storage at −70°C; 1.0%, Pre-30, PS with 1.0% of salt using pre-rigor muscle during batter storage at −30°C; 1.0%, Pre-70, PS with 1.0% of salt using pre-rigor muscle during batter storage at −70°C.

a Means having the different superscripts in the same column (treatment or storage time) are different (p<0.05).

**Table 6:** Cooking loss, expressible moisture, TBARS, and VBN values of pork sausages as affected by different salt level, rigor state and frozen conditions

Item		CL	EM	TBARS	VBN
Treatment^1)^					
Salt 1.5%	Post-30	29.1±1.90^b^	5.85±2.11^a^	0.26±0.01^a^	2.00±0.13^a^
	Post-70	30.6±2.55^ab^	6.80±1.30^a^	0.26±0.01^a^	2.02±0.15^a^
Salt 1.0%	Pre-30	31.2±1.87^ab^	7.07±2.53^a^	0.26±0.01^a^	2.02±0.12^a^
	Pre-70	31.7±2.87^a^	6.06±0.68^a^	0.25±0.01^a^	1.95±0.00^a^
Storage time (wks)					
0		6.49±1.49^a^	30.7±3.63^a^	0.26±0.01^a^	1.97±0.13^a^
4		6.37±1.73^a^	31.0±2.74^a^	0.26±0.01^a^	1.98±0.10^a^
8		6.26±1.24^a^	30.9±2.15^a^	0.26±0.01^a^	2.00±0.08^a^
12		6.66±2.70^a^	31.9±3.32^a^	0.26±0.01^a^	2.02±0.08^a^

CL, cooking loss; EM, expressible moisture; TBARS, thiobarbituric acid reactive substances; VBN, volatile basic nitrogen.

1) 1.5%, Post-30, pork sausage (PS) with 1.5% of salt using post-rigor muscle during batter storage at −30°C; 1.5%, Post-70, PS with 1.5% of salt using post-rigor muscle during batter storage at −70°C; 1.0%, Pre-30, PS with 1.0% of salt using pre-rigor muscle during batter storage at −30°C; 1.0%, Pre-70, PS with 1.0% of salt using pre-rigor muscle during batter storage at −70°C.

a,b Means having the different superscripts in the same column are different (p<0.05).
